# Supplementary figures and images for: Long-Term Transcriptional Activity at Zero Growth of a Cosmopolitan Rare Biosphere Member
Source: mBio. 2019 Feb 12;10(1):e02189-18. doi: 10.1128/mBio.02189-18 (PMC6372793; doi:10.1128/mBio.02189-18)

**Fig. S1.**

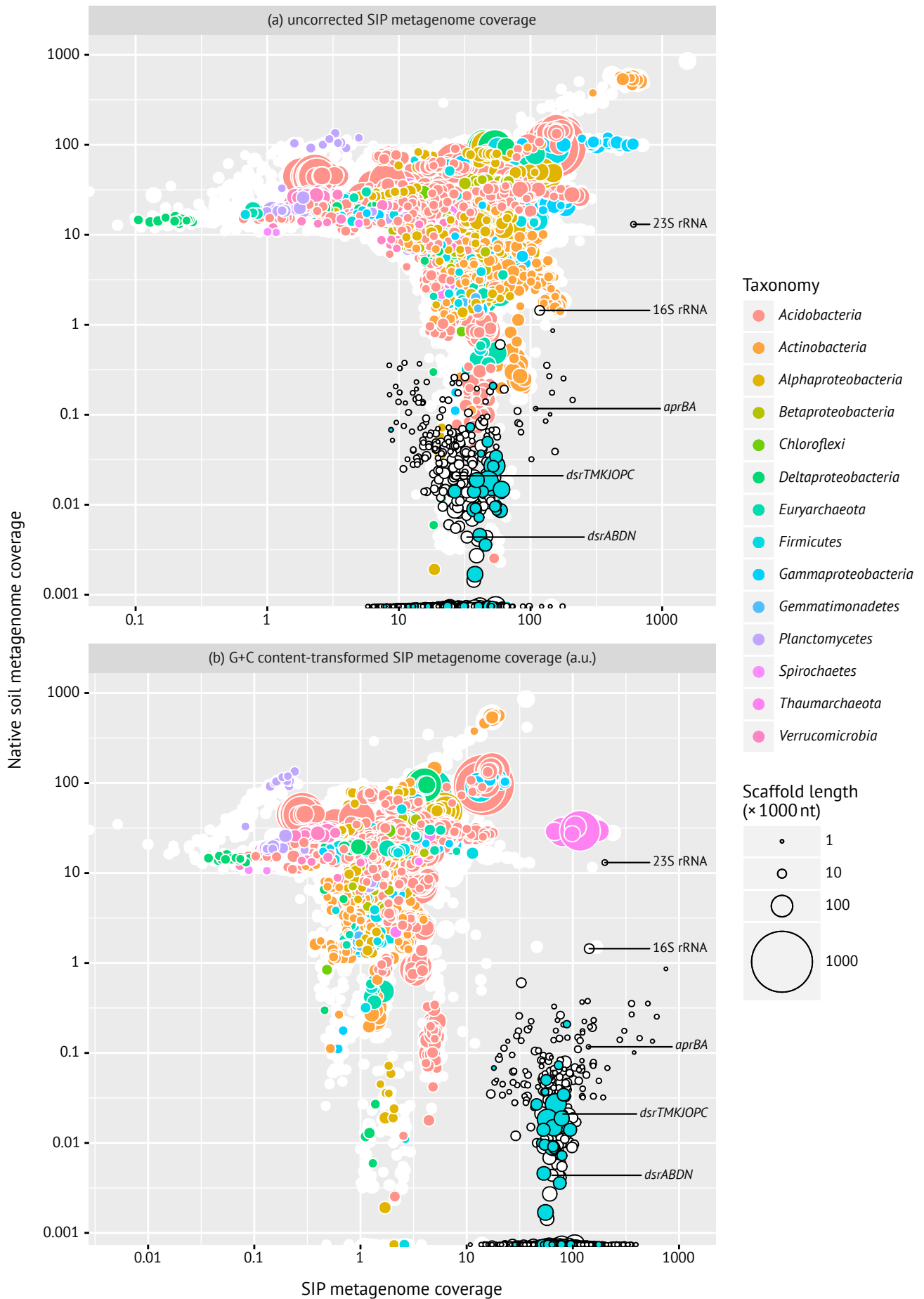

Supplement: FIG S1 [file mBio.02189-18-sf001.pdf]

# Fig. S2.

## (a) 16S rRNA gene

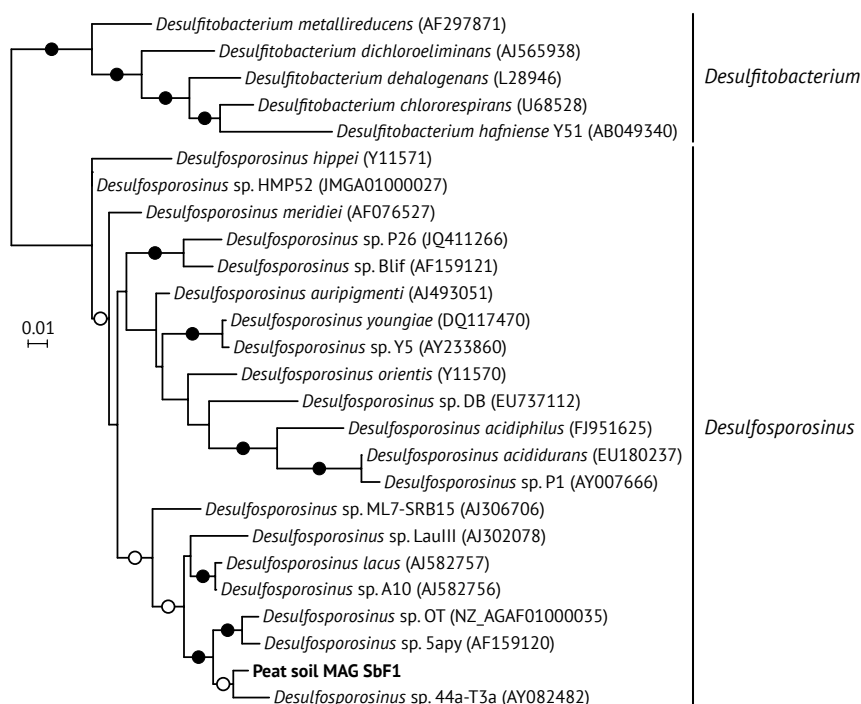

## (b) genome

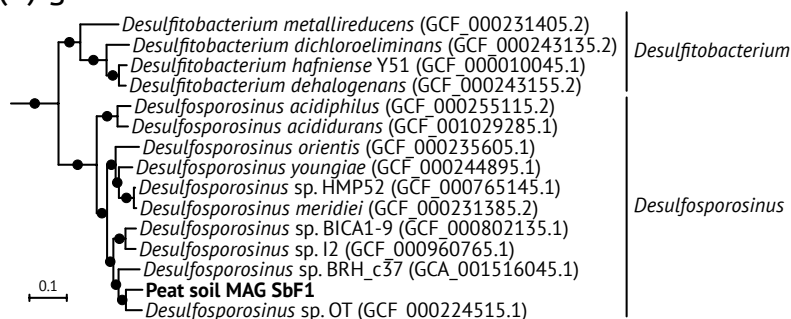

Supplement: FIG S2 [file mBio.02189-18-sf002.pdf]

**Fig. S3.**

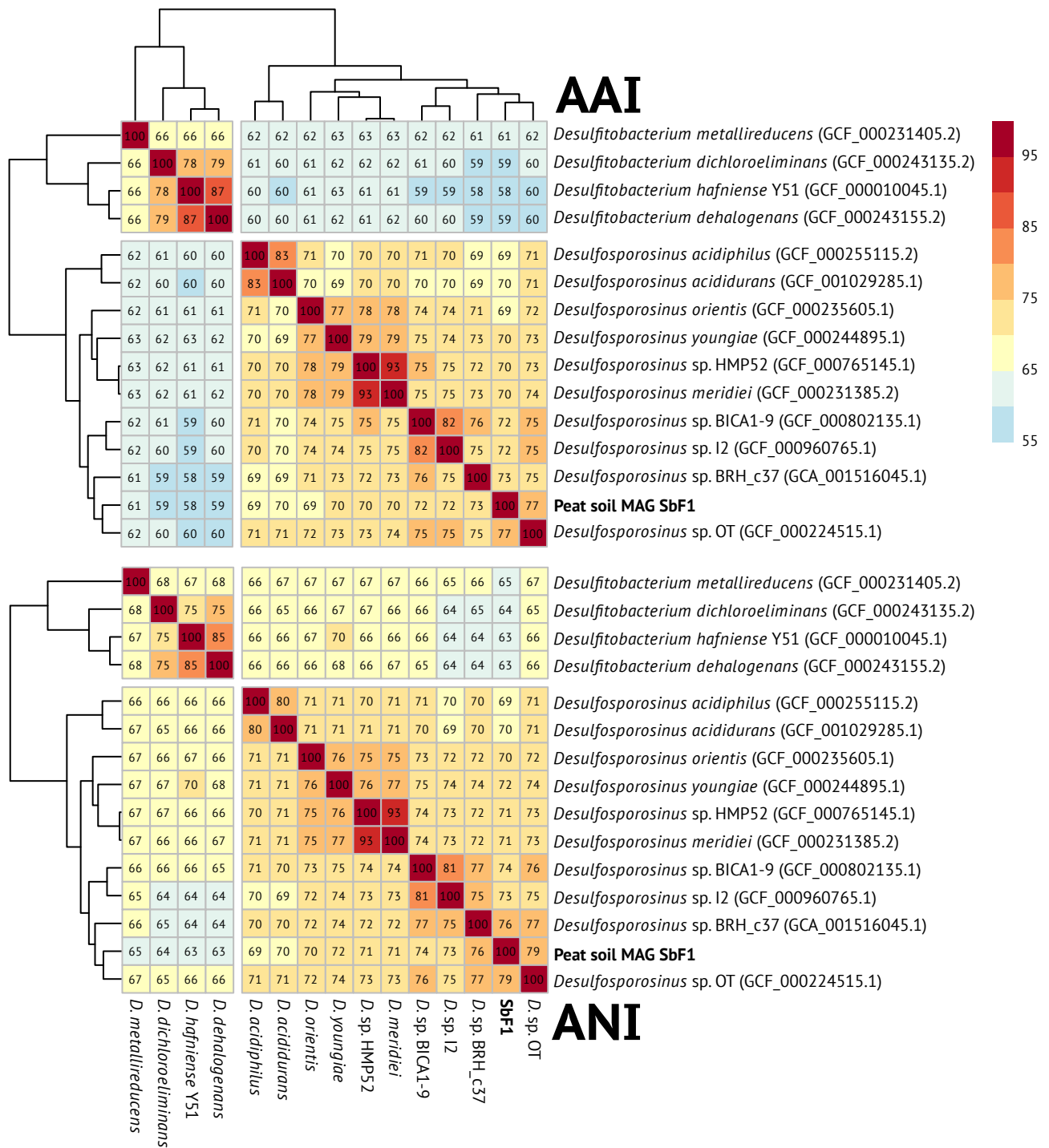

Supplement: FIG S3 [file mBio.02189-18-sf003.pdf]

Fig. S4.

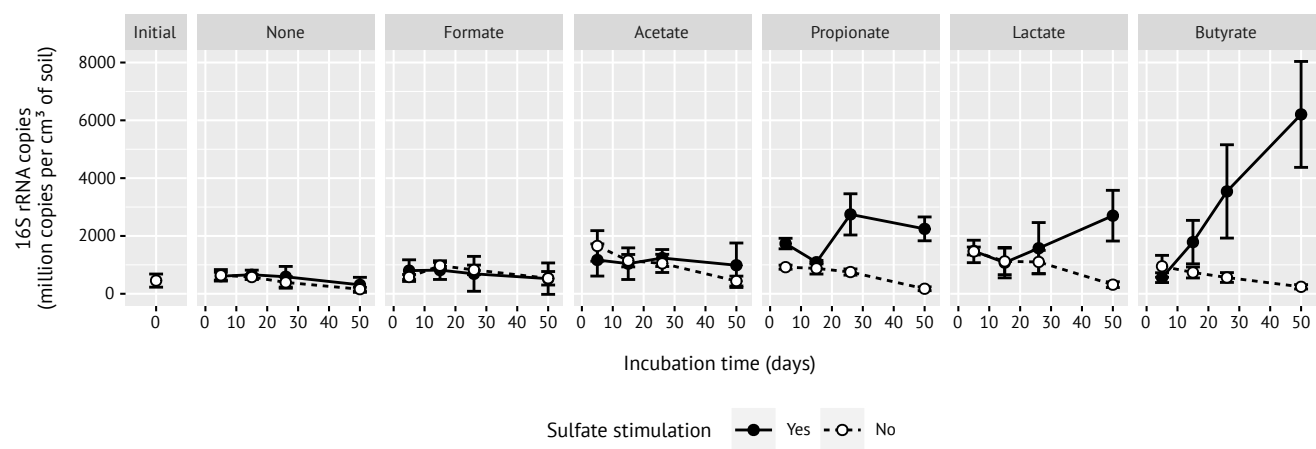

Supplement: FIG S4 [file mBio.02189-18-sf004.pdf]

Fig. S6.

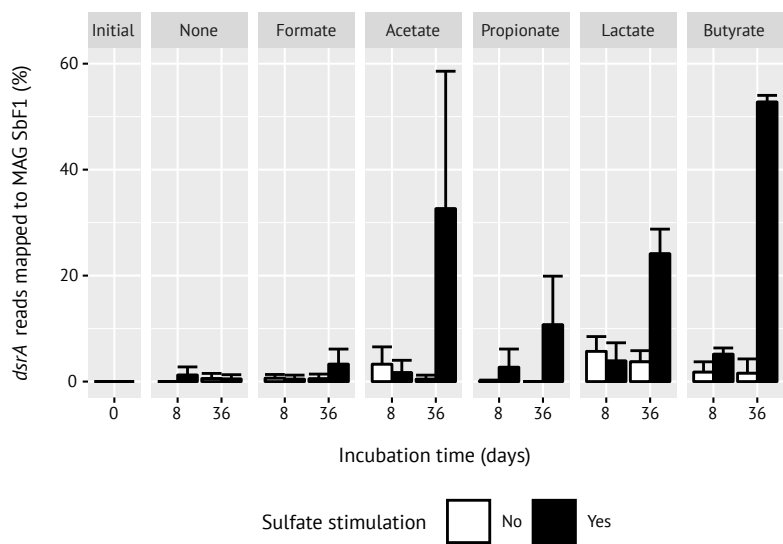

Supplement: FIG S6 [file mBio.02189-18-sf006.pdf]

Fig. S5.

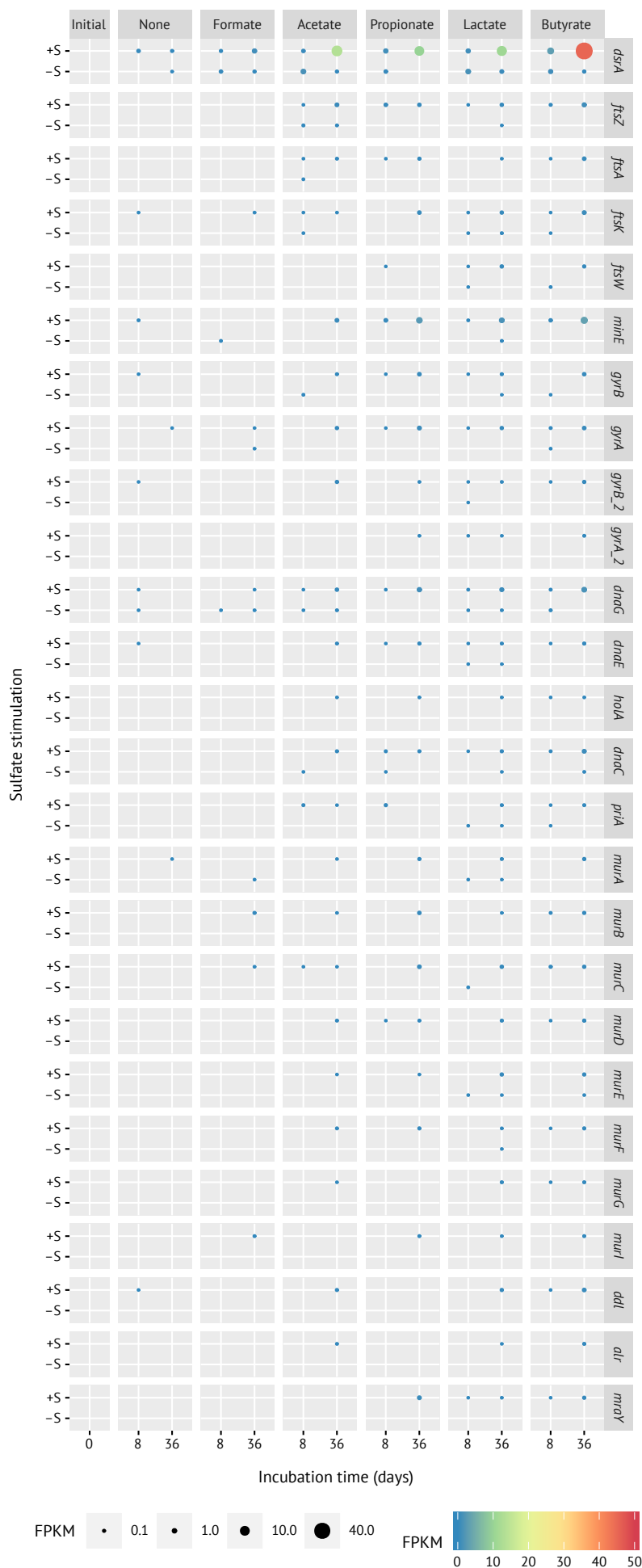

Supplement: FIG S5 [file mBio.02189-18-sf005.pdf]
