# Supplementary material for: Long-Term Transcriptional Activity at Zero Growth of a Cosmopolitan Rare Biosphere Member
Source: mBio. 2019 Feb 12;10(1):e02189-18. doi: 10.1128/mBio.02189-18 (PMC6372793; doi:10.1128/mBio.02189-18)
Supplement: TEXT S1 [file mBio.02189-18-s0001.pdf]

Long-term transcriptional activity at zero growth by a cosmopolitan rare biosphere member

Supplementary Information

Bela Hausmann, Claus Pelikan, Thomas Rattei, Alexander Loy, Michael Pester

Table of Contents

Supplementary Methods 1
  Calculation of minimum sulfate turnover for maintenance 1
Supplementary Tables 2
  Table S1 2
Supplementary Figures 3
  Fig. S1 3
  Fig. S2 3
  Fig. S3 3
  Fig. S4 3
  Fig. S5 3
  Fig. S6 4
Supplementary References 5

Supplementary Methods

Calculation of minimum sulfate turnover for maintenance

The minimum sulfate turnover required for maintenance was calculated according to the species-independent Arrhenius equation outlined in (1). Here, $m_e = Ae^{-Ea/RT}$ with m_e as the free energy consumption rate for zero growth, A as a constant factor for anaerobic microorganisms (4.99×10^{12} kJ g d.wt.$^{-1}$ d^{-1}), E_a as constant activation energy (69.4 kJ mol^{-1}), R as the universal gas constant (8.314 J mol^{-1} K^{-1}), and T as temperature in K. We used a temperature of 14 °C (288.15 K) for our calculations because this was the temperature at which the incubations were performed. The resulting m_e was converted to cell-specific sulfate reduction rates required for maintenance based on the energy yield of a sulfate-reducing microorganism when converting lactate to acetate (−226.4 kJ mol sulfate^{-1} at 14°C and pH 5, all reactants at standard concentrations) and a conversion factor of dry weight biomass to cell numbers of 2.9×10^{13} g d.wt. cell^{-1} (2).

Supplementary Tables

Table S1

(a) Summary of all genomic features in *Desulfosporosinus* sp. MAG SbF1. Genes encoding the energy metabolism or central cellular functions are given first. COG class IDs were assigned by MaGe (Cognitor, www.ncbi.nlm.nih.gov/COG/). bactNOG and NOG IDs were assigned by best-match principle (3, 4). Spearman's rank correlation is given for each gene's normalized transcript counts as compared to the sum of normalized mRNA counts (FDR-adjusted p-values are indicated by asterisks: *, < 0.05; **, < 0.01; ***, < 0.001). Expression clusters represent the clusters assigned by correlation and hierarchical clustering analysis. The next five columns are $\log_2$ fold-changes of expression levels after 36 days of incubation in the sulfate-stimulated microcosms (i.e., substrate vs no-substrate-control). Missing fold-changes are due to all counts being zero in both compared treatments. Ranks are based on mean fragments per kilobase per million total fragments (FPKM). Also here, only data of sulfate-stimulated microcosms after 36 days of incubation are shown in addition to the native soil. Missing ranks indicate that expression was never detected in any replicate. Fragmented, i.e., mainly incompletely assembled genes are indicated by _a, _b, and _c. A ^{1} or ^{2} in the strand column indicates that this CDS is either the first or last on a scaffold, respectively (depending on the reading frame). (b) Characteristics and coverage of all scaffolds belonging to *Desulfosporosinus* sp. MAG SbF1. The two scaffolds with the highest coverage encode the 23S and 16S rRNA genes, respectively. (c) Expression levels of selected CDS in the analysed anoxic peat soil microcosms given in FPKM (mean ± one standard deviation). Loci are sorted as in Table S1a. Headers display the individual treatments used in the peat soil microcosms: without and with external sulfate added; amended substrate; and days of incubation.

Supplementary Figures

Fig. S1

Differential coverage plots of assembled scaffolds with *Desulfosporosinus* sp. MAG SbF1 scaffolds highlighted by black circles. The average coverage per scaffold in the SIP metagenome is visualized without (a) and with (b) G+C content transformation (see Materials and Methods). Taxonomic affiliation is indicated by color and based on BLAST as described previously (5). White circles represent unclassified scaffolds. Only scaffolds >10 000 nt length are shown, except when belonging to SbF1. Scaffolds encoding selected genes in SbF1 are labelled accordingly.

Fig. S2

(a) Maximum likelihood 16S rRNA gene tree of species belonging to the genera *Desulfosporosinus* and *Desulfitobacterium*. Branch supports of ≥0.9 and ≥0.7 are indicated by filled and open circles, respectively. GenBank accession numbers are given in parentheses. (b) Bayesian inference phylogenomic tree showing the phylogenetic placement of *Desulfosporosinus* sp. MAG SbF1. All branches were supported >0.9 (filled circles). The tree was rooted against genomes from the *Acidobacteria*, *Proteobacteria*, and *Verrucomicrobia* (not shown). Genome accession numbers are given in parentheses.

Fig. S3

Two-way average amino and nucleic acid identities between *Desulfosporosinus* and *Desulfitobacterium* species genomes (in%, written into cells). The dendrogram is based on Fig. S2b.

Fig. S4

Time-resolved 16S rRNA copies of the low-abundance *Desulfosporosinus* population as determined by quantitative PCR, modified from (6). Error bars are ± one standard deviation (n=3; n=2 for propionate with sulfate stimulation, all days, and butyrate with sulfate stimulation, day 50).(6). Solid lines and symbols represent sulfate-stimulated microcosms whereas dashed lines and open symbols represent control microcosms without external sulfate. Panels represent the various substrate incubations, initial stands for initial peat soil.

Fig. S5

Time-resolved changes of all unambiguously identified genes related to cell division (*fts*Z, *fts*A, *fts*K, *fts*W, *min*E), DNA replication (*gyr*B, *gyr*A, *dna*G, *dna*E, *hol*A, *dna*C, *pri*A), and cell envelope biogenesis (*mur*ABCDEFGI, *ddl*, *alr*, *mra*Y, Table S1a); *dsr*A is included as reference, analogous to Fig. 3. Panels represent the various substrate incubations: initial, initial peat soil to set up peat microcosms; +/−S, incubations with or without external sulfate. The size and color of the dots represent average FPKM values of the respective normalized gene expression.

Fig. S6

Fraction of transcriptome reads mapped to the *dsr*A gene of *Desulfosporosinus* sp. MAG SbF1 relative to reads mapped to all publicly available *dsr*A gene sequences from this peatland (4, 7–9). Panels represent the various substrate incubations: initial, initial peat soil to set up peat microcosms. *dsr*A sequences were *de novo* aligned using MAFFT (10), then end-trimmed to remove underrepresented regions of the alignment. Transcriptomic reads were mapped on to the trimmed *dsr*A sequences with Bowtie 2 (11). Error bars represent one standard deviation of the mean (n=3).

Supplementary References

1. Harder J. 1997. Species-independent maintenance energy and natural population sizes. FEMS Microbiol Ecol 23:39–44.

2. Neidhardt FC, Ingraham JL, Schaechter M. 1990. Physiology of the bacterial cell: a molecular approach. Sinauer Associates, Sunderland, MA, USA.

3. Huerta-Cepas J, Szklarczyk D, Forslund K, Cook H, Heller D, Walter MC, Rattei T, Mende DR, Sunagawa S, Kuhn M, Jensen LJ, Mering C von, Bork P. 2016. eggNOG 4.5: a hierarchical orthology framework with improved functional annotations for eukaryotic, prokaryotic and viral sequences. Nucleic Acids Res 44:D286–D293.

4. Hausmann B, Pelikan C, Herbold CW, Köstlbacher S, Albertsen M, Eichorst SA, Glavina Del Rio T, Huemer M, Nielsen PH, Rattei T, Stingl U, Tringe SG, Trojan D, Wentrup C, Woebken D, Pester M, Loy A. 2018. Peatland *Acidobacteria* with a dissimilatory sulfur metabolism. ISME J.

5. Albertsen M, Hugenholtz P, Skarshewski A, Nielsen KL, Tyson GW, Nielsen PH. 2013. Genome sequences of rare, uncultured bacteria obtained by differential coverage binning of multiple metagenomes. Nat Biotechnol 31:533–538.

6. Hausmann B, Knorr K-H, Schreck K, Tringe SG, Glavina del Rio T, Loy A, Pester M. 2016. Consortia of low-abundance bacteria drive sulfate reduction-dependent degradation of fermentation products in peat soil microcosms. The ISME Journal 10:2365–2375.

7. Loy A, Küsel K, Lehner A, Drake HL, Wagner M. 2004. Microarray and functional gene analyses of sulfate-reducing prokaryotes in low-sulfate, acidic fens reveal cooccurrence of recognized genera and novel lineages. Appl Environ Microbiol 70:6998–7009.

8. Schmalenberger A, Drake HL, Küsel K. 2007. High unique diversity of sulfate-reducing prokaryotes characterized in a depth gradient in an acidic fen. Environ Microbiol 9:1317–1328.

9. Pester M, Bittner N, Deevong P, Wagner M, Loy A. 2010. A 'rare biosphere' microorganism contributes to sulfate reduction in a peatland. ISME J 4:1591–1602.

10. Katoh K, Standley DM. 2013. MAFFT multiple sequence alignment software version 7: improvements in performance and usability. Mol Biol Evol 30:772–780.

11. Langmead B, Salzberg SL. 2012. Fast gapped-read alignment with Bowtie 2. Nat Methods 9:357–359.
